# Supplementary material for: Prevalence of Undernutrition, Frailty and Sarcopenia in Community-Dwelling People Aged 50 Years and Above: Systematic Review and Meta-Analysis
Source: Nutrients. 2022 Apr 7;14(8):1537. doi: 10.3390/nu14081537 (PMC9032775; doi:10.3390/nu14081537)
Supplement: Supplementary file 1 [file nutrients-14-01537-s001.zip › Supplementary 1 Search strategy.pdf]

Supplementary 1 Search strategy

1. Geriatrics
2. Aged
3. 1 and 2
4. Malnutrition
5. Anorexia
6. Frail Elderly
7. Sarcopenia
8. 4 or 5 or 6 or 7
9. Prevalence
10. Incidence
11. Eating
12. Nutrients
13. Mortality
14. Morbidity
15. 9 or 10 or 11 or 12 or 13 or 14
16. 3 and 8 and 15
17. Limit 16 to humans
