# Supplementary material for: Prevalence of Undernutrition, Frailty and Sarcopenia in Community-Dwelling People Aged 50 Years and Above: Systematic Review and Meta-Analysis
Source: Nutrients. 2022 Apr 7;14(8):1537. doi: 10.3390/nu14081537 (PMC9032775; doi:10.3390/nu14081537)
Supplement: Supplementary file 1 [file nutrients-14-01537-s001.zip › Supplementary 2 Joanna Briggs Quality Assessment.pdf]

Supplementary 1 Joanna Briggs Quality Assessment

Table S1. Quality assessment using JBI tool for Cohort studies.

<table>
<tr><th>Author</th><th>Q 1</th><th>Q 2</th><th>Q 3</th><th>Q 4</th><th>Q 5</th><th>Q 6</th><th>Q 7</th><th>Q 8</th><th>Q 9</th><th>Q 10</th><th>Q 11</th></tr>
<tr><td rowspan="2">Carey, 2008 [84]</td><td>N</td><td>N/A</td><td>U</td><td>N</td><td>N</td><td>N/A</td><td>U</td><td>Y</td><td>N/A</td><td>N</td><td>Y</td></tr>
<tr><td colspan="11">Weak, the authors did not mention any criteria for inclusion and exclusion, beside they had their two groups from two different regions with different characteristic. Additionally, they did not stated number of people lost during the follow-up from both groups and they did not conducted any statistical test for data missing.</td></tr>
<tr><td rowspan="2">Wu, 2018 [73]</td><td>N/A</td><td>N/A</td><td>Y</td><td>N</td><td>N</td><td>N/A</td><td>Y</td><td>N/A</td><td>N/A</td><td>N/A</td><td>Y</td></tr>
<tr><td colspan="11">Medium, because the author did not pinpoint the confounding factors nor the test used.</td></tr>
<tr><td rowspan="2">Diniz, 2018 [74]</td><td>N/A</td><td>N/A</td><td>Y</td><td>Y</td><td>Y</td><td>N/A</td><td>Y</td><td>Y</td><td>Y</td><td>N</td><td>Y</td></tr>
<tr><td colspan="11">Medium, because the author did not address strategies to deal with incomplete data after the follow-up, beside, they did not identify the inclusion and exclusion criteria</td></tr>
<tr><td rowspan="2">Doi, 2018 [75]</td><td>N/A or N</td><td>N/A</td><td>Y</td><td>U</td><td>U</td><td>N/A</td><td>Y</td><td>Y</td><td>Y</td><td>Y</td><td>Y</td></tr>
<tr><td colspan="11">Medium, the author did not stated nor identify confounder, also, they did not identify the inclusion and exclusion criteria</td></tr>
<tr><td rowspan="2">Jung, 2014 [66]</td><td>N/A or N</td><td>N/A</td><td>Y</td><td>Y</td><td>Y</td><td>N/A</td><td>Y</td><td>Y</td><td>Y</td><td>Y</td><td>Y</td></tr>
<tr><td colspan="11">Strong, they only did not identify the inclusion and exclusion criteria</td></tr>
<tr><td rowspan="2">Lee, 2018 [67]</td><td>N/A</td><td>N/A</td><td>Y</td><td>Y</td><td>Y</td><td>N/A</td><td>Y</td><td>Y</td><td>U</td><td>Y</td><td>Y</td></tr>
<tr><td colspan="11">Strong</td></tr>
<tr><td rowspan="2">Legrand, 2013 [69]</td><td>N/A</td><td>N/A</td><td>Y</td><td>Y</td><td>Y</td><td>N/A</td><td>Y</td><td>N/A</td><td>N/A</td><td>N/A</td><td>Y</td></tr>
<tr><td colspan="11">Strong</td></tr>
<tr><td rowspan="2">Masel, 2010 [76]</td><td>N/A</td><td>N/A</td><td>Y</td><td>N</td><td>N</td><td>N/A</td><td>Y</td><td>Y</td><td>Y</td><td>Y</td><td>Y</td></tr>
<tr><td colspan="11">Medium, the author did not identify confounding factors and did not used any test to deal with them.</td></tr>
<tr><td rowspan="2">Mitoku, 2014 [85]</td><td>N/A</td><td>N/A</td><td>N</td><td>U</td><td>Y</td><td>N/A</td><td>N</td><td>Y</td><td>Y</td><td>N</td><td>N</td></tr>
<tr><td colspan="11">Weak, the author did not state the confounder factors and the exposure (frailty)/outcome were based on health insurance not measured during the follow up. Besides, the author did not stated any strategies to deal with loss of follow-up (death). Additionally, the confounder were adjusted without performing regression test.</td></tr>
<tr><td rowspan="2">Zheng, 2016 [77]</td><td>N/A</td><td>N/A</td><td>Y</td><td>U</td><td>Y</td><td>N/A</td><td>Y</td><td>Y</td><td>U</td><td>N</td><td>Y</td></tr>
<tr><td colspan="11">Medium, The author did adjusted the confounding factors without identifying it earlier. Beside they mentioned percentage of lost to follow-up without describing the reasons nor consider it in term of statistical. However, they describe their characteristics.</td></tr>
<tr><td rowspan="2">Yu, 2014 [81]</td><td>N/A</td><td>N/A</td><td>Y</td><td>U</td><td>Y</td><td>N/A</td><td>Y</td><td>Y</td><td>U</td><td>N</td><td>Y</td></tr>
<tr><td colspan="11">Medium, the authors did adjustment for the confounding factors, but did not identify it as factors. Also, the authors only mentioned the loss of follow-up in figure without any other details nor considering it in statistics.</td></tr>
<tr><td rowspan="2">Wu, 2016 [82]</td><td>N/A</td><td>N/A</td><td>Y</td><td>U</td><td>Y</td><td>N/A</td><td>Y</td><td>Y</td><td>N</td><td>N</td><td>Y</td></tr>
<tr><td colspan="11">Medium, The authors did not identify the confounding clearly but they consider it in statistical analysis. Beside they did not mention any loss/incomplete participants nor utilise strategy to deal with.</td></tr>
<tr><td rowspan="2">Theou, 2017 [68]</td><td>N/A</td><td>N/A</td><td>Y</td><td>N</td><td>N</td><td>N/A</td><td>Y</td><td>Y</td><td>Y</td><td>U</td><td>Y</td></tr>
<tr><td colspan="11">Strong</td></tr>
<tr><td rowspan="2">Sim, 2019 [86]</td><td>N/A</td><td>N/A</td><td>Y</td><td>N</td><td>N</td><td>N/A</td><td>Y</td><td>Y</td><td>Y</td><td>U</td><td>N</td></tr>
<tr><td colspan="11">Weak, the authors used participants recruited from previous studies without pinout the inclusion and exclusion criteria nor mentioning it briefly. In addition, they did not identify confounding factors</td></tr>
</table>

	and did not conducted analysis regard it. Also, they mentioned number of participants died during the follow-up but they did not used any statistical test for it.										
Robinson, 2008 [8]	N/A	N/A	Y	N	N	N/A	Y	Y	N/A	N/A	U
	Weak, the author did not mention the confounder nor strategy to deal with. Beside the authors did not conduct regression test										
Dallmeier, 2020 [80]	NA	N	Y	Y	Y	NA	Y	Y	U	U	Y
	Medium, they have not mentioned how they measured height and weight, type of scale, took the median of three reading.										
Das, 2020 [78]	NA	Y	Y	Y	Y	NA	Y	Y	N	N	Y
	Medium										
Lohman, 2020 [70]	NA	Y	Y	Y	Y	NA	Y	Y	U	U	Y
	Strong										
Lorenzo-lopes, 2019 [71]	NA	Y	Y	Y	Y	NA	Y	Y	Y	Y	Y
	Strong										
Murayama, 2020 [72]	NA	Y	Y	Y	Y	NA	Y	NA	NA	NA	Y
	Strong										
Nguyen, 2019 [79]	NA	Y	Y	Y	Y	NA	Y	U	U	N	Y
	Medium										

Y = Yes, U = Unclear, N = No and N/A = Not applicable.

Table S2. Quality assessment using JBI tool for cross-sectional studies.

<table>
<tr><th>Author</th><th>Q1</th><th>Q2</th><th>Q3</th><th>Q4</th><th>Q5</th><th>Q6</th><th>Q7</th><th>Q8</th></tr>
<tr><td rowspan="2">Ambagtsheer, 2019</td><td>Y</td><td>Y</td><td>Y</td><td>N/A</td><td>N</td><td>N</td><td>N/A</td><td>N</td></tr>
<tr><td colspan="8">Weak, it is a feasibility study and the authors did not count for confounder by identifying it nor strategies to deal with. Beside they did not include any regression nor stratification test.</td></tr>
<tr><td rowspan="2">González-Pichard, 2013</td><td>N</td><td>Y</td><td>Y</td><td>Y</td><td>Y</td><td>Y</td><td>Y</td><td>Y</td></tr>
<tr><td colspan="8">Strong</td></tr>
<tr><td rowspan="2">Buffa, 2010</td><td>N</td><td>Y</td><td>Y</td><td>N/A</td><td>U</td><td>N</td><td>Y</td><td>Y</td></tr>
<tr><td colspan="8">Medium, because the author did not mention any details about inclusion and exclusion criteria. It was not clear if the author discus the confounder factors.</td></tr>
<tr><td rowspan="2">Curcio, 2014</td><td>Y</td><td>Y</td><td>Y</td><td>Y</td><td>Y</td><td>Y</td><td>Y</td><td>Y</td></tr>
<tr><td colspan="8">Strong</td></tr>
<tr><td rowspan="2">Coqueiro, 2017</td><td>Y</td><td>Y</td><td>Y</td><td>Y</td><td>Y</td><td>Y</td><td>Y</td><td>Y</td></tr>
<tr><td colspan="8">Strong</td></tr>
<tr><td rowspan="2">Albuquerque Sous, 2012</td><td>Y</td><td>Y</td><td>Y</td><td>Y</td><td>N</td><td>N</td><td>Y</td><td>Y</td></tr>
<tr><td colspan="8">Medium, the author did not state the confounder factors, however the study is about factors associated with frailty. Yet the confounder factors cannot build into association nor relation.</td></tr>
<tr><td rowspan="2">El-Sherbiny, 2016</td><td>N</td><td>Y</td><td>Y</td><td>Y</td><td>U</td><td>Y</td><td>Y</td><td>Y</td></tr>
<tr><td colspan="8">Medium, the author did not stated clear criteria for inclusion and exclusion, beside, the authors did not identify the confounding factors although they mention confounding effect and test to be conducted.</td></tr>
<tr><td rowspan="2">Gao, 2015</td><td>Y</td><td>Y</td><td>Y</td><td>Y</td><td>U</td><td>Y</td><td>Y</td><td>Y</td></tr>
<tr><td colspan="8">Medium, the authors did not identify the confounding factors although they mention covariates and conducted regression test</td></tr>
<tr><td rowspan="2">Jung, 2016</td><td>Y</td><td>Y</td><td>Y</td><td>Y</td><td>N</td><td>N</td><td>Y</td><td>N</td></tr>
<tr><td colspan="8">Medium, the authors did not identify the confounding factors and did not address it by conducted regression/stratification tests</td></tr>
<tr><td rowspan="2">Kabir, 2006</td><td>N</td><td>Y</td><td>Y</td><td>Y</td><td>N</td><td>N</td><td>Y</td><td>U</td></tr>
<tr><td colspan="8">Weak, the authors did not identify inclusion/exclusion criteria and the confounding factors. However, they conducted regression model but did not state for 22/factors etc</td></tr>
<tr><td rowspan="2">Kurkcu, 2017</td><td>U</td><td>Y</td><td>N</td><td>Y</td><td>Y</td><td>Y</td><td>Y</td><td>Y</td></tr>
<tr><td colspan="8">Weak, as the method used in categorising the frailty was not validated, beside the author did not identify the criteria for inclusion/exclusion participants in details</td></tr>
<tr><td rowspan="2">Mori, 2019</td><td>N</td><td>Y</td><td>Y</td><td>Y</td><td>Y</td><td>Y</td><td>Y</td><td>Y</td></tr>
<tr><td colspan="8">Strong, the author did not identify inclusion and exclusion criteria also the confounder. However they used stratifying by age and sex</td></tr>
<tr><td rowspan="2">Nanri, 2018</td><td>U</td><td>Y</td><td>Y</td><td>Y</td><td>N</td><td>N</td><td>Y</td><td>N</td></tr>
<tr><td colspan="8">Medium, the author did not mention confounding nor strategy to deal with. Also, they did not conduct regression test nor stratification</td></tr>
<tr><td rowspan="2">Parra-Rodríguez, 2016</td><td>Y</td><td>Y</td><td>Y</td><td>Y</td><td>N</td><td>N</td><td>Y</td><td>N</td></tr>
<tr><td colspan="8">Medium, the author did not count for confounder by identifying nor by statistical testing</td></tr>
<tr><td rowspan="2">Perez-Zepeda, 2019</td><td>Y</td><td>Y</td><td>Y</td><td>Y</td><td>Y</td><td>Y</td><td>Y</td><td>Y</td></tr>
<tr><td colspan="8">Strong (however the used FI deficit that was created and validated by Mexican study previously. They did not used the international index)</td></tr>
<tr><td rowspan="2">Yamanashi, 2016</td><td>Y</td><td>Y</td><td>Y</td><td>Y</td><td>Y</td><td>Y</td><td>Y</td><td>Y</td></tr>
<tr><td colspan="8">Strong</td></tr>
<tr><td rowspan="2">Wilhelm-Leen, 2013</td><td>Y</td><td>Y</td><td>Y</td><td>Y</td><td>N</td><td>Y</td><td>Y</td><td>Y</td></tr>
<tr><td colspan="8">Strong</td></tr>
</table>

Tramontano, 2017	Y	Y	Y	Y	Y	Y	Y	Y
	Strong							
Pegorari, 2014	Y	Y	Y	Y	U	U	Y	Y
	Medium, the authors did not specify the confounding factors and not mentioning strategy used for it.							
Santos-Eggimann, 2009	Y	Y	Y	Y	Y	Y	Y	Y
	Strong							
Reis Junior, 2014	N	Y	Y	Y	U/Y	Y	Y	Y
	Strong							
Huang, 2020	N	Y	Y	Y	N	N	Y	N
	Low, the author did not mention confounder, did not even address it in statistic. Also, the author did not identify inclusion and exclusion criteria							
Rivas-Ruiz, 2019	Y	Y	Y	Y	Y	U	Y	Y
	Strong							
Xu, 2020	Y	Y	Y	Y	N	Y	Y	Y
	Strong							

Y = Yes, U = Unclear, N = No and N/A = Not applicable.
