# Supplementary material for: Prevalence of Undernutrition, Frailty and Sarcopenia in Community-Dwelling People Aged 50 Years and Above: Systematic Review and Meta-Analysis
Source: Nutrients. 2022 Apr 7;14(8):1537. doi: 10.3390/nu14081537 (PMC9032775; doi:10.3390/nu14081537)
Supplement: Supplementary file 1 [file nutrients-14-01537-s001.zip › Supplementary 3 Prevalence Forest Plots.pdf]

Supplementary 3 Prevalence Forest Plots

Figure S1. (**A**) Prevalence of undernutrition analysis split by gender in the older people living in the community; (**B**) Prevalence of undernutrition analysis split by tools in the older people living in the community; (**C**) Prevalence of frailty analysis split by gender in the older people living in the community (Das include only in male, because all participants were male. There are eight studies not included in the forest plots because they did not report frailty status according to gender (15, 43, 47, 48, 50, 51, 61, 72)); (**D**) Prevalence of frailty analysis split by tools in the older people living in the community; (**E**) Prevalence of sarcopenia analysis split by gender in the older people living in the community; (**F**) Prevalence of sarcopenia analysis split by tools in the older people living in the community; ES = Effect size, CI = Confidence interval, M = Male, F = Female.

(**A**)

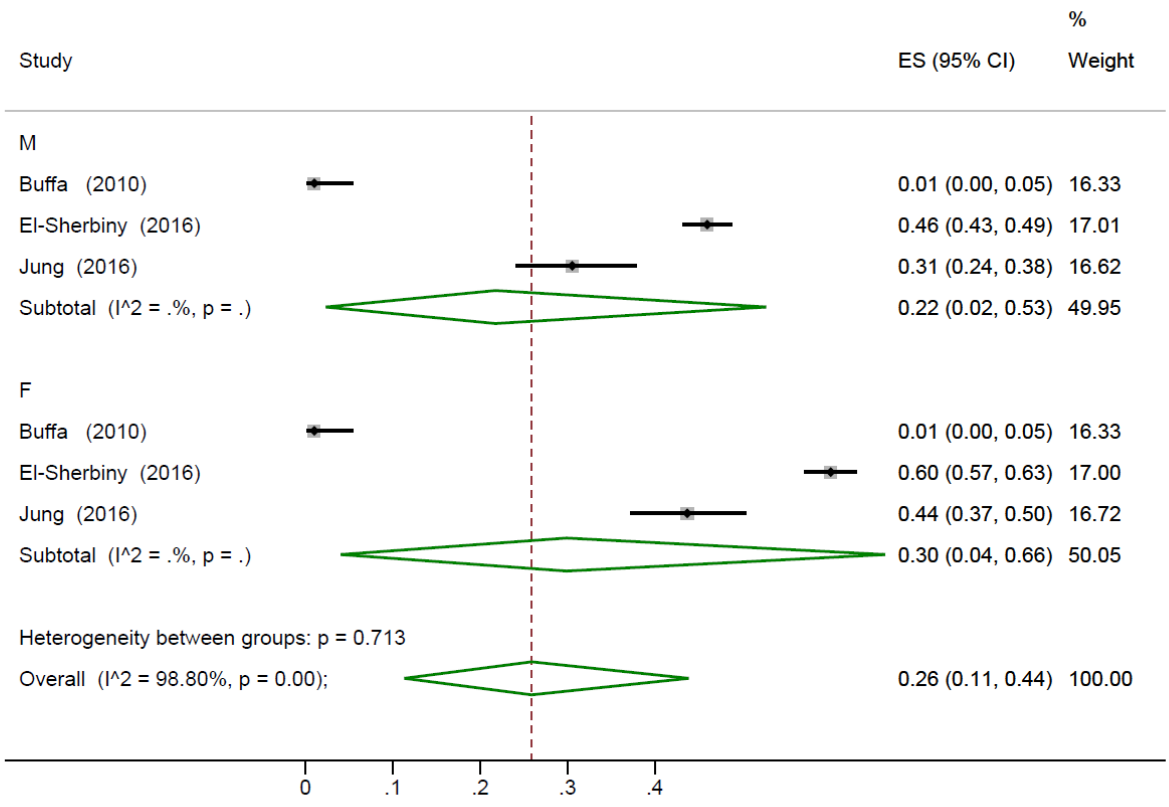

(**B**)

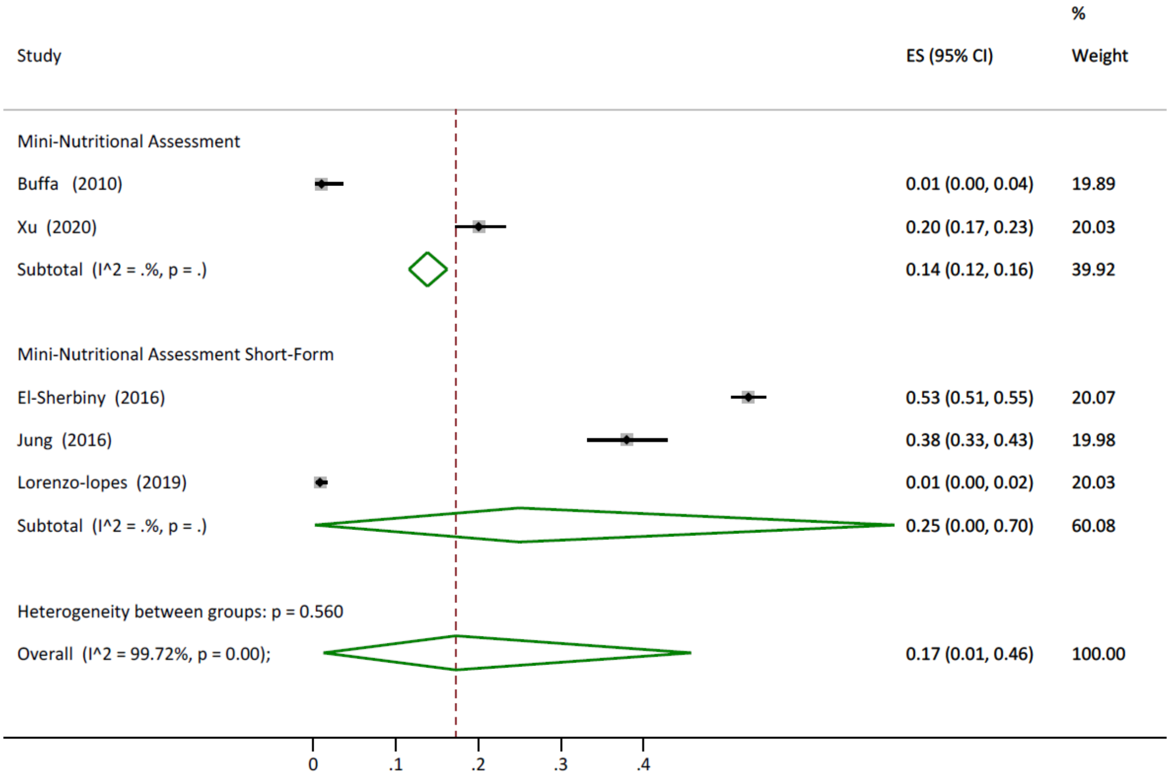

Study
ES (95% CI)
%
Weight
Mini-Nutritional Assessment
Buffa (2010)
0.01 (0.00, 0.04)
19.89
Xu (2020)
0.20 (0.17, 0.23)
20.03
Subtotal (I^2 = .%, p = .)
0.14 (0.12, 0.16)
39.92
Mini-Nutritional Assessment Short-Form
El-Sherbiny (2016)
0.53 (0.51, 0.55)
20.07
Jung (2016)
0.38 (0.33, 0.43)
19.98
Lorenzo-lopes (2019)
0.01 (0.00, 0.02)
20.03
Subtotal (I^2 = .%, p = .)
0.25 (0.00, 0.70)
60.08
Heterogeneity between groups: p = 0.560
Overall (I^2 = 99.72%, p = 0.00);
0.17 (0.01, 0.46)
100.00
0
.1
.2
.3
.4

(**C**)

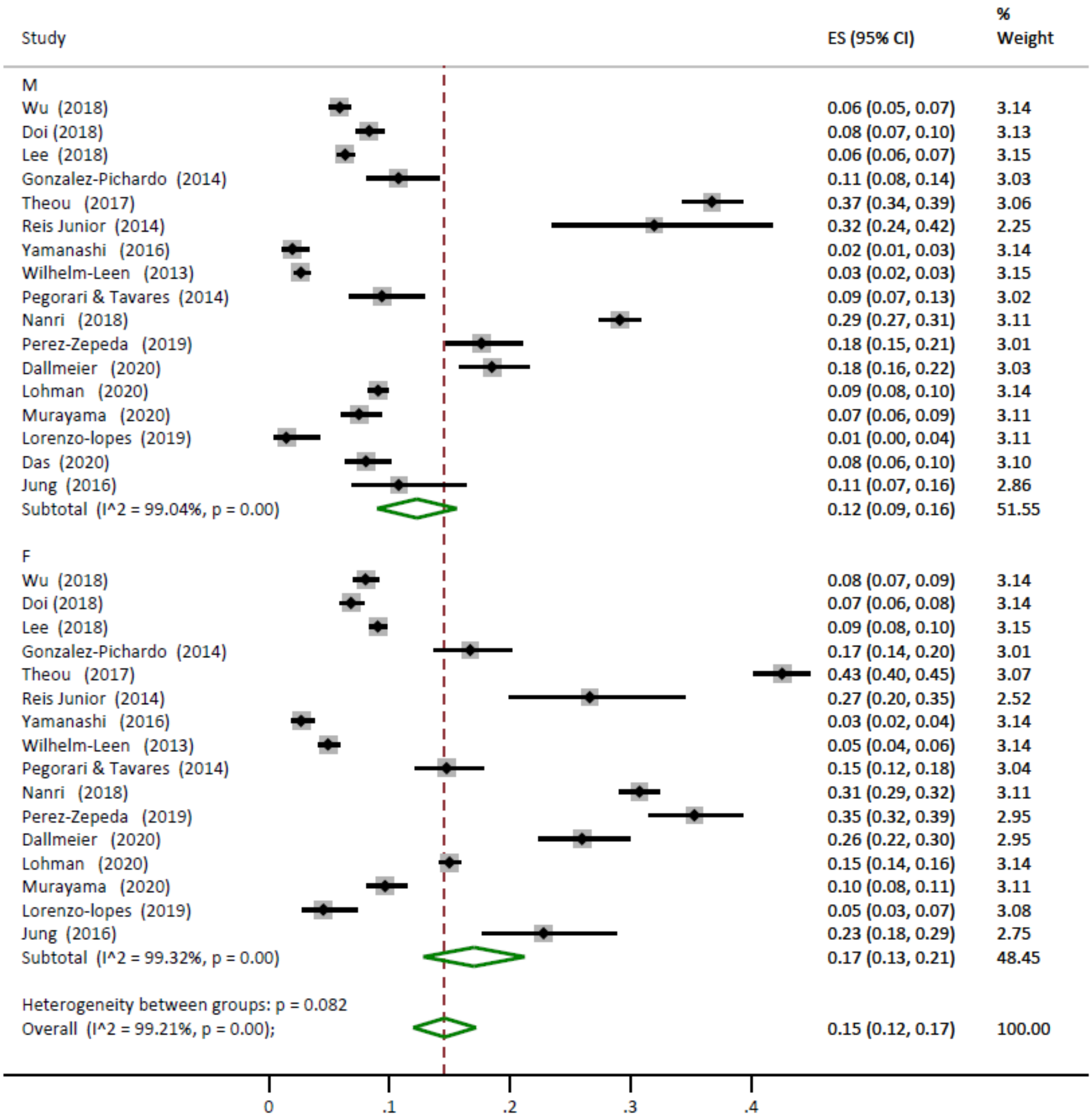
Study
ES (95% CI)
% Weight
M
Wu (2018) 0.06 (0.05, 0.07) 3.14
Doi (2018) 0.08 (0.07, 0.10) 3.13
Lee (2018) 0.06 (0.06, 0.07) 3.15
Gonzalez-Pichardo (2014) 0.11 (0.08, 0.14) 3.03
Theou (2017) 0.37 (0.34, 0.39) 3.06
Reis Junior (2014) 0.32 (0.24, 0.42) 2.25
Yamanashi (2016) 0.02 (0.01, 0.03) 3.14
Wilhelm-Leen (2013) 0.03 (0.02, 0.03) 3.15
Pegorari & Tavares (2014) 0.09 (0.07, 0.13) 3.02
Nanri (2018) 0.29 (0.27, 0.31) 3.11
Perez-Zepeda (2019) 0.18 (0.15, 0.21) 3.01
Dallmeier (2020) 0.18 (0.16, 0.22) 3.03
Lohman (2020) 0.09 (0.08, 0.10) 3.14
Murayama (2020) 0.07 (0.06, 0.09) 3.11
Lorenzo-lopes (2019) 0.01 (0.00, 0.04) 3.11
Das (2020) 0.08 (0.06, 0.10) 3.10
Jung (2016) 0.11 (0.07, 0.16) 2.86
Subtotal (I^2 = 99.04%, p = 0.00) 0.12 (0.09, 0.16) 51.55
F
Wu (2018) 0.08 (0.07, 0.09) 3.14
Doi (2018) 0.07 (0.06, 0.08) 3.14
Lee (2018) 0.09 (0.08, 0.10) 3.15
Gonzalez-Pichardo (2014) 0.17 (0.14, 0.20) 3.01
Theou (2017) 0.43 (0.40, 0.45) 3.07
Reis Junior (2014) 0.27 (0.20, 0.35) 2.52
Yamanashi (2016) 0.03 (0.02, 0.04) 3.14
Wilhelm-Leen (2013) 0.05 (0.04, 0.06) 3.14
Pegorari & Tavares (2014) 0.15 (0.12, 0.18) 3.04
Nanri (2018) 0.31 (0.29, 0.32) 3.11
Perez-Zepeda (2019) 0.35 (0.32, 0.39) 2.95
Dallmeier (2020) 0.26 (0.22, 0.30) 2.95
Lohman (2020) 0.15 (0.14, 0.16) 3.14
Murayama (2020) 0.10 (0.08, 0.11) 3.11
Lorenzo-lopes (2019) 0.05 (0.03, 0.07) 3.08
Jung (2016) 0.23 (0.18, 0.29) 2.75
Subtotal (I^2 = 99.32%, p = 0.00) 0.17 (0.13, 0.21) 48.45
Heterogeneity between groups: p = 0.082
Overall (I^2 = 99.21%, p = 0.00); 0.15 (0.12, 0.17) 100.00
0
.1
.2
.3
.4

(**D**)

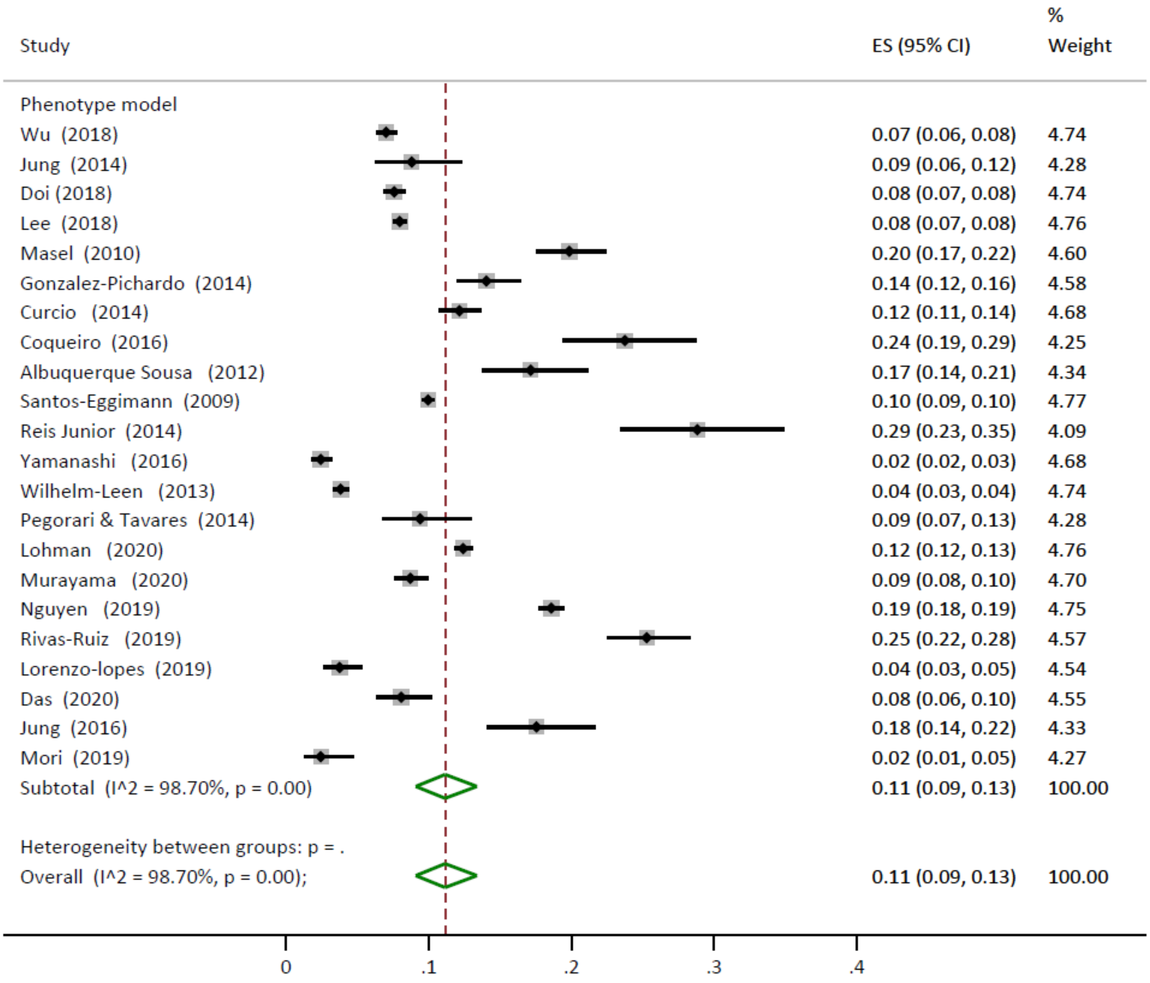
Study
ES (95% CI)
% Weight
Phenotype model
Wu (2018) 0.07 (0.06, 0.08) 4.74
Jung (2014) 0.09 (0.06, 0.12) 4.28
Doi (2018) 0.08 (0.07, 0.08) 4.74
Lee (2018) 0.08 (0.07, 0.08) 4.76
Masel (2010) 0.20 (0.17, 0.22) 4.60
Gonzalez-Pichardo (2014) 0.14 (0.12, 0.16) 4.58
Curcio (2014) 0.12 (0.11, 0.14) 4.68
Coqueiro (2016) 0.24 (0.19, 0.29) 4.25
Albuquerque Sousa (2012) 0.17 (0.14, 0.21) 4.34
Santos-Eggimann (2009) 0.10 (0.09, 0.10) 4.77
Reis Junior (2014) 0.29 (0.23, 0.35) 4.09
Yamanashi (2016) 0.02 (0.02, 0.03) 4.68
Wilhelm-Leen (2013) 0.04 (0.03, 0.04) 4.74
Pegorari & Tavares (2014) 0.09 (0.07, 0.13) 4.28
Lohman (2020) 0.12 (0.12, 0.13) 4.76
Murayama (2020) 0.09 (0.08, 0.10) 4.70
Nguyen (2019) 0.19 (0.18, 0.19) 4.75
Rivas-Ruiz (2019) 0.25 (0.22, 0.28) 4.57
Lorenzo-lopes (2019) 0.04 (0.03, 0.05) 4.54
Das (2020) 0.08 (0.06, 0.10) 4.55
Jung (2016) 0.18 (0.14, 0.22) 4.33
Mori (2019) 0.02 (0.01, 0.05) 4.27
Subtotal (I^2 = 98.70%, p = 0.00) 0.11 (0.09, 0.13) 100.00
Heterogeneity between groups: p = .
Overall (I^2 = 98.70%, p = 0.00); 0.11 (0.09, 0.13) 100.00
0
.1
.2
.3
.4

(**E**)

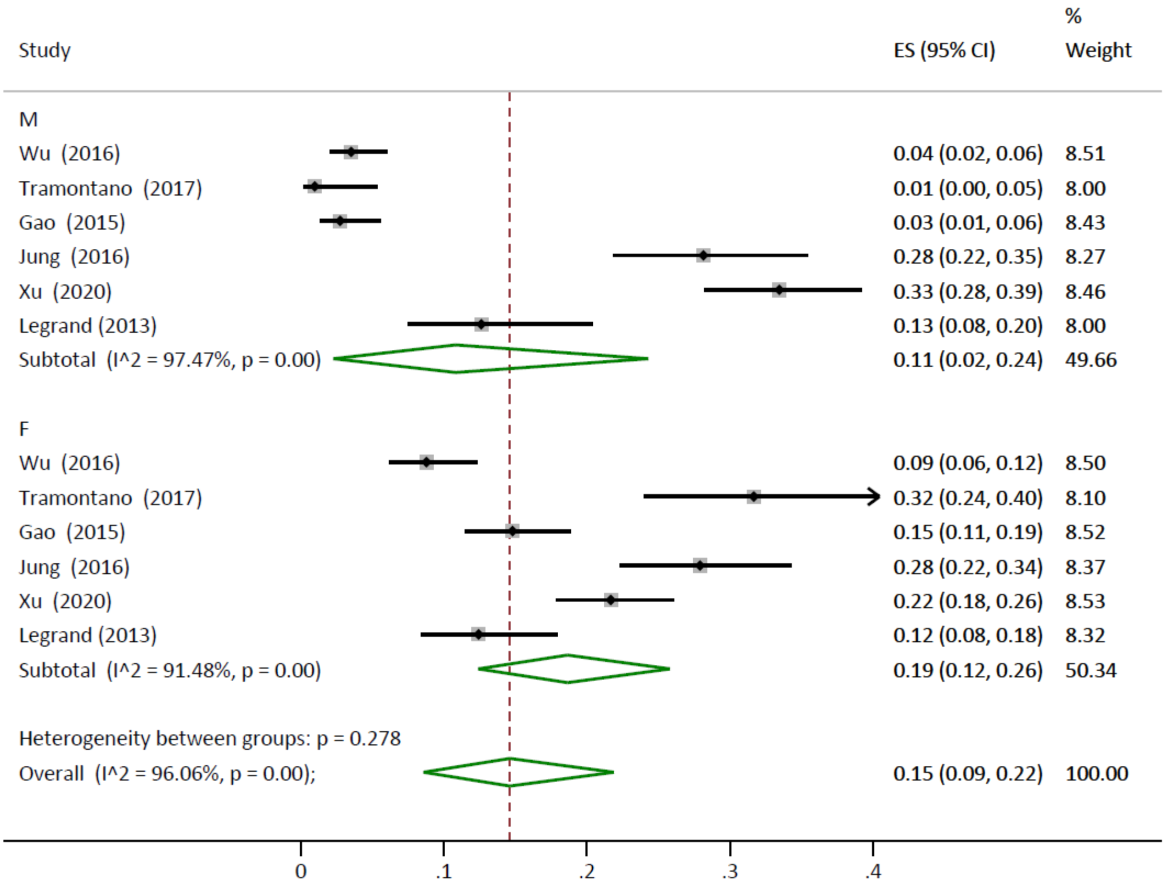
Study
ES (95% CI)
% Weight
M
Wu (2016) 0.04 (0.02, 0.06) 8.51
Tramontano (2017) 0.01 (0.00, 0.05) 8.00
Gao (2015) 0.03 (0.01, 0.06) 8.43
Jung (2016) 0.28 (0.22, 0.35) 8.27
Xu (2020) 0.33 (0.28, 0.39) 8.46
Legrand (2013) 0.13 (0.08, 0.20) 8.00
Subtotal (I^2 = 97.47%, p = 0.00) 0.11 (0.02, 0.24) 49.66
F
Wu (2016) 0.09 (0.06, 0.12) 8.50
Tramontano (2017) 0.32 (0.24, 0.40) 8.10
Gao (2015) 0.15 (0.11, 0.19) 8.52
Jung (2016) 0.28 (0.22, 0.34) 8.37
Xu (2020) 0.22 (0.18, 0.26) 8.53
Legrand (2013) 0.12 (0.08, 0.18) 8.32
Subtotal (I^2 = 91.48%, p = 0.00) 0.19 (0.12, 0.26) 50.34
Heterogeneity between groups: p = 0.278
Overall (I^2 = 96.06%, p = 0.00); 0.15 (0.09, 0.22) 100.00
0
.1
.2
.3
.4

(**F**)

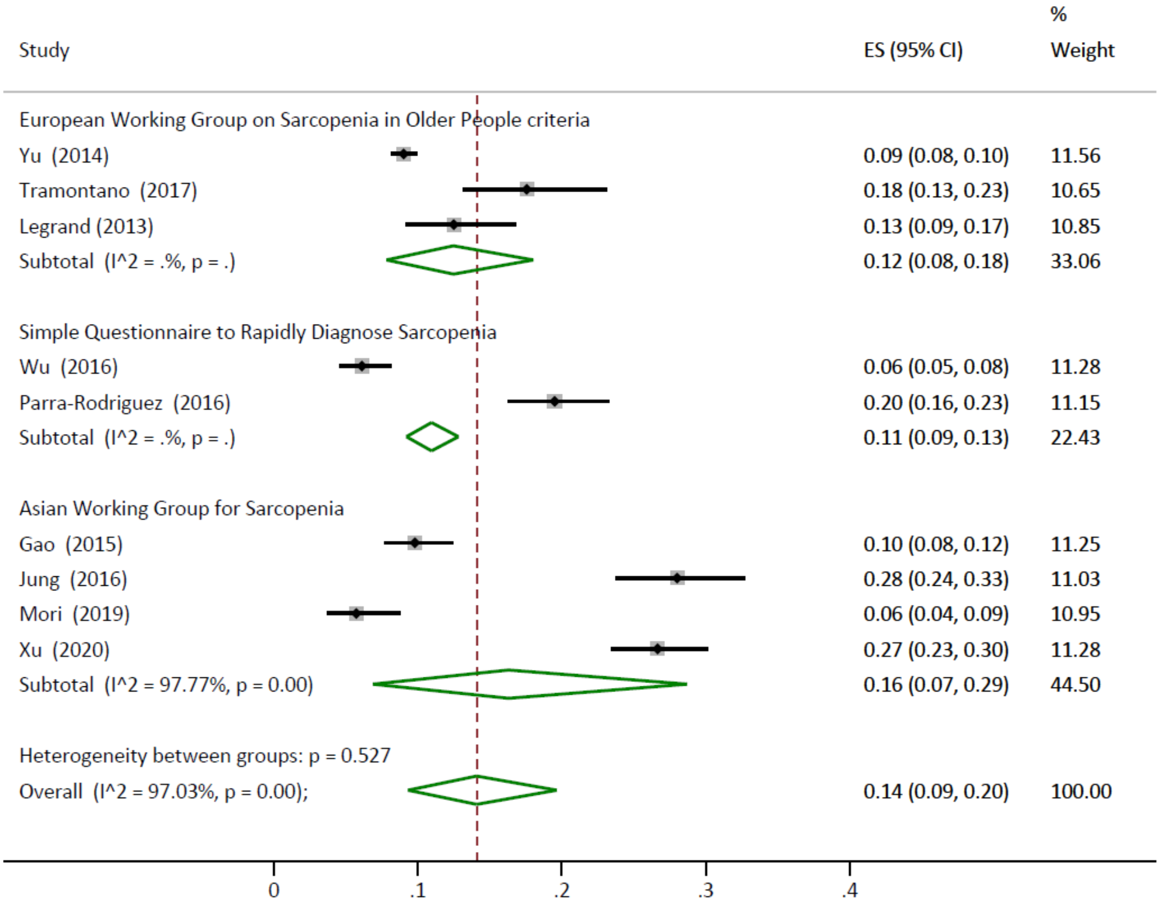
Study
ES (95% CI)
% Weight
European Working Group on Sarcopenia in Older People criteria
Yu (2014) 0.09 (0.08, 0.10) 11.56
Tramontano (2017) 0.18 (0.13, 0.23) 10.65
Legrand (2013) 0.13 (0.09, 0.17) 10.85
Subtotal (I^2 = .%, p = .) 0.12 (0.08, 0.18) 33.06
Simple Questionnaire to Rapidly Diagnose Sarcopenia
Wu (2016) 0.06 (0.05, 0.08) 11.28
Parra-Rodriguez (2016) 0.20 (0.16, 0.23) 11.15
Subtotal (I^2 = .%, p = .) 0.11 (0.09, 0.13) 22.43
Asian Working Group for Sarcopenia
Gao (2015) 0.10 (0.08, 0.12) 11.25
Jung (2016) 0.28 (0.24, 0.33) 11.03
Mori (2019) 0.06 (0.04, 0.09) 10.95
Xu (2020) 0.27 (0.23, 0.30) 11.28
Subtotal (I^2 = 97.77%, p = 0.00) 0.16 (0.07, 0.29) 44.50
Heterogeneity between groups: p = 0.527
Overall (I^2 = 97.03%, p = 0.00); 0.14 (0.09, 0.20) 100.00
0
.1
.2
.3
.4
