# Supplementary material for: Prevalence of Undernutrition, Frailty and Sarcopenia in Community-Dwelling People Aged 50 Years and Above: Systematic Review and Meta-Analysis
Source: Nutrients. 2022 Apr 7;14(8):1537. doi: 10.3390/nu14081537 (PMC9032775; doi:10.3390/nu14081537)
Supplement: Supplementary file 1 [file nutrients-14-01537-s001.zip › Supplementary 4 Study characteristics.pdf]

# Supplementary 4 Study Characteristics

| Author (year) | Country | Study design | Setting(Population) | Main findings |
|---|---|---|---|---|
| | | | Malnutrition | |
| Buffa (2010) [65] | Italy | cross-sectional | Orroli (central Sardinia, Italy) | With respect to their contemporaries from other regions, the elderly of Orroli presented a better nutritional status, a similar worsening with age and generally higher sexual dimorphism |
| El-Sherbiny (2016) [63] | Egypt | cross-sectional | Older people in Fayoum governorate | Non-communicable disease, malnutrition and depression were prevalent in our older population. These findings indicated the need for comprehensive integrated medical, psychological and nutritional health care at the level of the primary health care units. |
| | | | Frailty | |
| Wu (2017) [73] | China | Cohort | From the China Health & Retirement Longitudinal Study | We found 7.0% of adults aged 60 years or older were frail. Frailty is more prevalent at advanced ages, among women, and persons with low education. Age-adjusted frailty prevalence ranged from 3.3% in the Southeast and the Northeast to 9.1% in the Northwest, and was more than 1.5 times higher in rural versus urban areas. Frail versus nonfrail persons had higher prevalence of comorbidities, falls, disability, and functional limitation. |
| Diniz (2018) [74] | Brazil | cohort | Older adults living in the city of Ribeirão Preto, located in the northwest region of the state of São Paulo, Brazil. | Verified that older people were considered non-frail in 2007/2008 (59.5%); 22.9% were identified as apparently vulnerable, and 17.6% were frail. In 2013, 28.7% were considered non-frail, 20.9% apparently vulnerable; and 50.4% were frail (an increase of 186%). A total of 24.7% died during the follow-up period; 45.7% were considered frail according to the Edmonton Frail Scale. The survival analysis showed that frail older people were more likely to die |
| Doi (2018) [75] | Japan | cohort | Taken from the Obu Study of Health Promotion for the Elderly, which is part of the National Center for Geriatrics and Gerontology - Study of Geriatric Syndromes | The results of the present prospective study provide key information on the transitional status of frailty and the risk factors for progression to frailty. A further study is required to determine the pathophysiological changes that underlie the transition to frailty. |
| Jung (2014) [66] | Korea | cohort | Older people living in Seongnam, Gyeongi Province | This prospective cohort study revealed the association between frailty status and subsequent decline in lean mass in community-dwelling elderly people. The results of this study may provide evidence to help determine the mechanism of the harmful cycle of the frailty syndrome |
| Lee (2018) [67] | Korea | cohort | A nationally representative sample of community-dwelling Koreans | Frailty was a significant predictor of 3-year mortality in community-dwelling older adults, with the association being moderated by baseline cognitive status. Taking cognitive function into account may allow better prediction of adverse outcomes of frailty in later life. |

| | | | | |
|---|---|---|---|---|
| Masel (2010) [76] | United State | cohort | The Hispanic Established Populations for Epidemiologic Study of the Elderly | Older Mexican Americans identified as frail experienced decreased survival in this sample than their prefrail or nonfrail counterparts. Reduced survival in persons categorized as frail was attenuated by adjusting for the physical component of health related quality of life |
| Zheng (2016) [77] | China | cohort | Community in Beijing, China. | Frailty is more common for urban and female residents in the oldest old group. Being frail significantly predicts geriatric adverse outcomes, indicating the importance of early screening and intervention in frail individuals in primary care |
| Gonzales-Pichardo (2013) [47] | Mexican | cross-sectional | The Mexican Study of Nutritional and Psychosocial Markers of Frailty | Poor SRHS shares common correlates as well as health-related adverse outcomes with frailty syndrome, and remains associated with it even when possible confounders are taken into account. Therefore, poor SRHS could be further explored as an option for frailty syndrome screening |
| Theou (2017) [68] | United State | cohort | US National Health and Nutrition Examination Survey | The effect of sedentary behaviours on mortality varied by level of frailty. Adults with the highest frailty level experienced the greatest adverse impact. Low frailty levels (frailty index score ≤ 0.1) seemed to eliminate the increased risk of mortality associated with prolonged sitting, even among people who did not meet recommended physical activity guidelines |
| Curcio (2014) [48] | South America | cross-sectional | Older population in a rural area in the Andes Mountains | Relevant number of elderly persons living in rural areas in the Andes Mountains are frail. The prevalence of frailty is similar to that reported in other populations in the Latin American region. Our results support the use of modified Cardiovascular Health Study criteria to measure frailty in communities other than urban settings. Frailty in this study was strongly associated with comorbidities, and frailty and comorbidity predicted disability |
| Coqueiro (2017) [49] | Brazil | cross-sectional | Epidemiologic population-based household survey | Reducing the time with sedentary behavior and increasing the time with physical activity may prevent frailty syndrome. |
| Albuquerque (2012) [64] | Brazil | cross-sectional | Part of a multicenter project on Frailty in Elderly Brazilians (REDE FIBRA) | The factors associated with frailty suggest a predictive model that helps in understanding the syndrome, guiding actions that minimize adverse effects in the aging process. |
| Santos-Eggimann (2009) [15] | European Union (10 countries: Sweden, Denmark, Netherlands, Germany, Austria, Switzerla | cross-sectional | SHARE is a multidisciplinary European Union research project | A higher prevalence of frailty in southern countries is consistent with previous fi ndings of a north – south gradient for other health indicators in SHARE. Our data suggest that socioeconomic factors like education contribute to these differences in frailty and prefrailty |

| | | | | |
|---|---|---|---|---|
| | nd, France, Italy, Spain, Italy, Spain, Greece) | | | |
| ReisJunior (2014) [55] | Brazil | cross-sectional | Part of epidemiological study with a community and population basis | The evidence presented in this study demonstrates more variables associated with the frailty condition, reinforcing the concept of a multifactorial clinical syndrome that may result in the loss of functionality. |
| Yamanashi (2016) [52] | Japan | cross-sectional | Japanese national medical check-up in 2015. | Our study shows that multiple somatic symptoms are independently associated with frailty. Using more than 2 multiple somatic symptoms as a pre-screening tool for frailty may be appropriate |
| Wilhelm-Leen (2013) [53] | USA | cross-sectional | Third National Health and Nutritional Examination Survey (1988–1994) | Narrow phase angle is associated with frailty and mortality independent of age and comorbidity. |
| Pegorari (2014) [58] | Brazil | cross-sectional | Older individuals living in the urban area | Pre-frailty and frailty conditions presented a percentage higher than that reported by Brazilian studies and are associated with health-related variables. These variables can be prevented with interventions directed to the health of elderly individuals |
| Nanri (2018) [60] | Japan | cross-sectional | From Kameoka City, Kyoto, Japan | The higher dietary protein intake may be inversely associated with the prevalence of comprehensive frailty in Japanese men and women. Future studies are needed to examine associations of dietary protein intake within KCL domains. |
| Perez-Zepeda (2019) [51] | Mexico city | cross-sectional | The 2012 wave of the Mexican Health and Aging Study | Our findings show that an incremental association of abnormal biomarkers increases the probability of frailty, accounting for the multidimensional nature of frailty and the possible interplay between components of the system that potentiate to give rise to a negative condition such as frailty |
| Dallmeier (2020) [80] | Germany | cohort | The ActiFE Ulm (Activity and Function in the Elderly in Ulm) study is a population-based cohort study in community-dwelling older people (≥65 years) randomly selected in Ulm and | We observed a statistically significant difference for the age-adjusted eFI and the frailty prevalence in men and women. However, our analysis does not suggest the presence of effect modification by sex in the association with mortality. |

| | | | | |
|---|---|---|---|---|
| | | | adjacent regions in Southern Germany | |
| Lohman (2020) [70] | USA | cohort | Data for this study came from two linked sources: the Health and Retirement Study (HRS) and the National Death Index (NDI). We obtained permission to use the restricted HRS-NDI linked database through a data access agreement with the University of Michigan, and all analyses were performed in a secure virtual data enclave | Significantly greater risk of mortality from several different causes should be considered alongside the potential costs of screening and intervention for frailty in subspecialty and general geriatric clinical practice. Findings may help investigators estimate the potential impact of frailty reduction approaches on mortality. |
| Murayama (2020) [72] | japan | cohort | This study used data from the National Survey of the JapaneseElderly (NSJE) in 201 | This study provides important evidence on the prevalence of frailty in older Japanese people andfound substantial disparities by sociodemographic characteristics, health conditions, and geographical regions |
| Nguyen (2019) [79] | USA | cohort | Data are from the first five yearly rounds of NHATS, starting in 2011. The NHATS is a survey of a nationally representative sample of Medicare beneficiaries aged 65 years and older | Considering both multimorbidity patterns and frailty is important for identifying older adults at greater risk of mortality. Of the five patterns identified, the neuropsychiatric class was associated with lower survival across all frailty levels. |
| Rivas-Ruiz (2019) [57] | Spain | cross sectional | All participants should live within the community, be functionally independent (Barthel's Index > 90 points), be aged 70 years or more and provide signed informed consent | In this population of community-dwelling persons aged 70 years and over, the prevalence of frailty was 26%. Factors correlated with frailty were female sex, comorbidity, poorer self perceived lifestyle and health status, and dissatisfaction with the domestic environment. |

| | | | | |
|---|---|---|---|---|
| Sarcopenia | | | | |
| Yu (2014) [81] | China | cohort | Community-dwelling older Chinese adults | Sarcopenia incidence increases with age, but is potentially reversible in a Chinese elderly population. High body mass index is protective against sarcopenia incidence and its reversibility. Increasing physical activity and maintaining a healthy weight could be beneficial in the prevention of sarcopenia |
| Wu (2016) [82] | Taiwan | cohort | Community hospital in Taiwan | Sarcopenia screened using SARC-F was associated with subsequent QOL, overall hospitalization, overall emergency care use, and 4-year mortality. SARC-F can serve as a quick screening tool of sarcopenia |
| Tramontano (2017) [54] | South America | cross-sectional | Older adults of the Peruvian Andes | The prevalence of sarcopenia seems to be quite high among community-dwelling older subjects in the Peruvian Andes. Age, female sex, a low body mass index, little physical activity, a poor Six-Minute Walking Test scores, and a low number of children could be associated with this condition. |
| Legrand (2013) [69] | Belgium | cross-sectional | From The BELFRAIL study (BFC80+) was designed as a prospective, observational, population-based cohort study to evaluate subjects aged 80 years and older living in Belgium. | In a population-based sample of the very old the prevalence of sarcopenia according to the EWGSOP algorithm is similar to the prevalence of sarcopenia with SMI as a single criterion. A large number of participants with a sufficient SMI value showed low muscle strength and/or a poor SPPBm score. A low SPPBm was associated with grip strength but not with SMI. |
| Combination of two or more conditions | | | | |
| Xu (2020) [56] | China | cross sectional | We used advertising strategy (displaying posters) to recruit volunteers in the Beijisi community, Haidian District, Beijing in eastern China using a convenience sampling approach | We found over a quartile of the community-dwelling oldest old in China had sarcopenia. Older age, lower BMI, and worse nutritional status were significantly associated with the presence of sarcopenia. Sarcopenia was independently associated with disability and poor physical function. |
| Gao (2015) [62] | China | cross-sectional | Urban and rural communities in western China | Rural elders are more vulnerable to sarcopenia than urban elders in a sample of western China's elderly population. More attention should focus on rural populations in future sarcopenia studies. |
| Jung (2016) [61] | Korea | cross-sectional | residing in rural communities of Korea between October 2014 and December 2014 | Older Koreans living in rural communities have a significant burden of frailty and geriatric conditions that increase the risk of functional decline, poor quality of life, and mortality. The current study provides a basis to guide public health professionals and policy-makers in prioritizing certain areas of care and designing effective public health interventions to promote healthy aging of this vulnerable population. |

| | | | | |
|---|---|---|---|---|
| Mori (2019) [50] | Japan | cross-sectional | recruited for older adults aged 60-to 85-years-old, at six community center (Kakogawa, Harima, Inami, Kato, Nishiwaki, Yokawa) from Harima community in Hyogo, Japan | This study showed that sarcopenia with frailty was had higher incidences of recurrent fall and poor HRQOL than robust older adults. Aging and poor energy, protein, and vitamin D intake, may be relevant factors for sarcopenia with frailty. |
| Parra-Rodriguez (2016) [59] | Mexico city | cross-sectional | The FraDySMex study, a 2-round evaluation of community-dwelling adults from 2 municipalities in Mexico City | The SARC-F scale was successfully adapted to Spanish language and validated in community-dwelling Mexican older adults |
| Lorenzo-lopes (2019) [71] | spain | cohort | Community-dwelling older people aged 65 and over who participated in the Effectiveness of the Comprehensive Gerontological Assessment and longitudinal follow-up in the healthy aging promotion (VERISAÚDE) | Our results confirm the dynamic of frailty and the bidirectional nature of frailty transitions, andindicate the need for preventing and treating these conditions in later life in order to minimize the burden offrailty. |
| Das (2020) [78] | Austriala | cohort | The Concord Health and Ageing in Men Project (CHAMP) is a longitudinal study of aging in men | Poor antioxidant intake, particularly vitamin E, is a plausible factor associated with incident frailty among older men. This supports the need for clinical trials of diets rich in antioxidants or possibly low-dose antioxidant supplements, for prevention of frailty. |
