# Supplementary material for: Prevalence of Undernutrition, Frailty and Sarcopenia in Community-Dwelling People Aged 50 Years and Above: Systematic Review and Meta-Analysis
Source: Nutrients. 2022 Apr 7;14(8):1537. doi: 10.3390/nu14081537 (PMC9032775; doi:10.3390/nu14081537)
Supplement: Supplementary file 1 [file nutrients-14-01537-s001.zip › Supplementary 5 Studies included in this systematic review presented with female to male ratio.pdf]

Supplementary 5. Table Studies included in this systematic review presented with female to male ratio.

Study author	Sex ratio	Study author	Sex ratio
(Wu et al., 2017) [73]	1	(Nanri et al., 2018) [60]	0.9
(Diniz et al., 2018) [74]	0.5	(Perez-Zepeda et al., 2019) [51]	0.9
(Jung et al., 2014) [66]	1.1	(Dallmeier et al., 2020) [80]	1.3
(Doi et. Al, 2018) [75]	0.9	(Lohman et al., 2020) [70]	0.6
(Lee et al., 2018) [67]	0.6	(Murayama et al., 2020) [72]	0.6
(Masel et al., 2010) [76]	0.5	(Nguyen et al., 2019) [79]	0.7
(Zheng et al., 2016) [77]	0.6	(Rivas-Ruiz et al.., 2019) [57]	0.8
(Gonzalez-Pichardo et al, 2013) [47]	0.8	(Lorenzo-lopes et al., 2019) [71]	0.6
(Theou et al., 2017) [68]	0.8	(Das et al., 2020)* [78]	
(Curcio et al., 2017) [48]	0.9	(Buffa et al., 2010) [65]	1
(Coqueiro et al., 2017) [49]	0.9	(El-Sherbiny et al., 2016) [63]	1.1
(Albuquerque Sousa et al., 2012) [64]	0.6	(Yu et al., 2014) [81]	1
(Santos-Eggimann et al., 2009)* [15]		(Wu et al., 2016) [82]	1
(Reis Junior et al., 2014) [55]	0.6	(Tramontano et al., 2017) [54]	0.8
(Yamanashi et al, 2016) [52]	0.5	(Gao et al., 2015) [62]	0.7
(Wilhelm-Leen et al, 2013) [53]	0.9	(Jung et al., 2016) [61]	0.7
(Pegorari & Tavares, 2014) [58]	0.5	(Parra-Rodriguez et al., 2016) [59]	0.2
(Mori et al., 2019) [50]	0.3	(Xu et al., 2020) [56]	0.7

*Santos study did not have information about the numbers of females and males. Das study had males only.
